# Development and validation of the Japanese version of cognitive flexibility scale

**DOI:** 10.1186/s13104-016-2070-y

**Published:** 2016-05-17

**Authors:** Keiko Oshiro, Sawako Nagaoka, Eiji Shimizu

**Affiliations:** Research Centre for Child Mental Development, Graduate School of Medicine, Chiba University, 1-8-1 Inohana, Chuo-ku, Chiba, 260-8670 Japan; United Graduate School of Child Development, Osaka University, Kanazawa University, Hamamatsu University School of Medicine, Chiba University and University of Fukui, 1-6 Yamadaoka, Suita, Osaka, Japan; Department of Cognitive Behavioral Physiology, Chiba University Graduate School of Medicine, 1-8-1 Inohana, Chuo-ku, Chiba, 260-8670 Japan

**Keywords:** Cognitive flexibility, Cognitive flexibility scale, Scale development, Japanese version, Reliability, Validity

## Abstract

**Background:**

Various instruments have been developed to assess cognitive flexibility, which is an important construct in psychology. Among these, the self-report cognitive flexibility scale (CFS) is particularly popular for use with English speakers; however, there is not yet a Japanese version of this scale. This study reports on the development of a Japanese version of the cognitive flexibility scale (CFS-J), and the assessment of its internal consistency, test–retest reliability, and validities.

**Methods:**

We used the standard translation–back-translation process to develop the Japanese wording of the items and tested these using a sample of 335 eligible participants who did not have a mental illness, were aged 18 years or older, and lived in the suburbs of Tokyo. Participants included office workers, public servants, and college students; 71.6 % were women and 64.8 % were students. The translated scale’s internal consistency reliability was assessed by calculating Cronbach’s alpha and McDonald’s omega, and test–retest reliability was assessed with 107 eligible participants via intra-class correlation coefficient (ICC) and Spearman’s correlation of coefficient. Exploratory factory analysis (EFA) and correlations with other scales were used to examine the factor-based and concurrent validities of the CFS-J.

**Results:**

Results indicated that the CFS-J has good internal consistency (Cronbach’s alpha = 0.847, McDonald’s omega = 0.871) and acceptable test–retest reliability (Spearman’s = 0.687, ICC = 0.689). EFA provided evidence that the CFS-J has a one-factor structure and factor loadings were generally appropriate. The total CFS-J score was significantly and positively correlated with the cognitive flexibility inventory-Japanese version and its two subscales, along with the cognitive control scale and the positive subscale of the short Japanese version of the automatic thought questionnaire–revised (ATQ-R); further, it had a significantly negative correlation with the negative subscale of the ATQ-R (*p*s < 0.001). This study developed a Japanese version of the cognitive flexibility scale and confirmed its reliability and validity among a sample of people with no current mental illness, who were living in the suburbs of Tokyo.

## Background

Cognitive flexibility is one aspect of executive functioning that encompasses the ability to produce diverse ideas, consider response alternatives, and modify behaviors to manage changing circumstances [1]. The most popular definition appears to be “the readiness with which the person’s concept system changes selectively in response to appropriate environmental stimuli” [2], and Spiro and Jehng [3] defined cognitive flexibility, in their cognitive flexibility theory, as “the ability to adaptively re-assemble diverse elements of knowledge to fit the particular needs of a given understanding or problem-solving situation,” and Johnco [1] stated that “cognitive flexibility is likely to be an important mental ability to facilitate the learning of cognitive restructuring as a skill to increase adaptive functioning and the ability to adjust to changes in life circumstances.”

Many instruments have been developed to assess cognitive flexibility, including performance-based measures, such as the Wisconsin card sorting test [4] and the Stroop color and word test [5]. These “task-oriented” instruments generally require ponderous processing by administrators, and are difficult for applying to large groups or for continuous implementation in clinical situations; thus, self-reported measures are more practical owing to their brevity and easy administration. Two self-reported questionnaires have been developed to assess cognitive flexibility: the cognitive flexibility scale (CFS) [6] and the cognitive flexibility inventory (CFI) [7]. Because the CFS was developed earlier than the CFI, a number of subsequent studies have utilized the former instrument for assessing cognitive flexibility, and reported the usefulness of the CFS for assessing the severity of various psychiatric disorders in clinical situations, e.g., generalized anxiety disorder [8], depression and anxiety disorders [3, 9], eating disorders [10], and posttraumatic stress disorder [11, 12].

The above evidence suggests that cognitive flexibility could also be a useful measure of the outcomes of psychiatric treatment. However, there is no existing Japanese language version of the CFS, which has been used as the popular instrument for measuring cognitive flexibility especially in the English-speaking world; therefore, in this study we translated this scale into Japanese. We also conducted an initial exploration of the internal consistency reliability, test–retest reliability, and concurrent validity (including factor-based validity) of the Japanese version of the CFS (CFS-J) among people with no existing mental illness and who were living in the suburbs of Tokyo.

## Methods

### Translation process

We translated the CFS into Japanese with the permission of the original author [6], then conducted a back-translation into English to preserve its correspondence with the original scale, other than allowing for linguistic and cultural differences. Six experts with backgrounds in psychiatry (two), psychiatric nursing (one), and clinical psychology (three), who were native Japanese speakers with a good command of English, translated the 12 CFS items into Japanese. A translation company performed the Japanese to English back-translation, and a bilingual English literature professor who was not given any information about the CFS checked for correspondence of meaning between the translated and original versions of the scale. Then, the back-translated version was sent to the author of the CFS to check for agreement of meaning with the original scale, with comments provided on problematic items. We repeated the translation and back-translation procedures until the author approved the correspondence between all the items in the English and Japanese versions, and this version was examined in the study described below. These translation and back-translation processes follow the usual guidelines for cross-cultural scale adaptations [13].

### Participants

The sample size of 300 was targeted according to the recommended procedure [14] for structural equation modeling sampling sufficiency (n:q = 10:1 ratio of number of subjects to number of test items) [15]. Participants were volunteers recruited from October 2014 to February 2015 by the authors and their assistants, and included office workers at private companies, public school teachers, and junior college and university students living in the suburbs of Tokyo. An explanation of the aims and implications of the study and data collection procedures, including information that non-participants would not be disadvantaged, was provided on the cover page of the questionnaire, and 389 participants that consented to take part in the study submitted informed consent and completed the questionnaires. None of the participants were undergoing regular treatment for a mental illness at a hospital. Of the 389 eligible participants, we excluded 54 because they gave one or more missing responses or apparently unreliable responses (e.g., filling the same response number for all items). We used data from the remaining 335 (86.1 % of the initial sample) participants for further analyses. Furthermore, a subsample (n = 168) of the eligible participants was asked to complete the same version of the CFS-J one to 2 weeks later to examine the scale’s test–retest reliability, and valid data from both data collections were obtained from 107 respondents. This study was approved by the ethics committee of the Graduate School of Medicine at Chiba University (receipt number 1894).

### Instruments

#### Cognitive flexibility scale-Japanese version (CFS-J)

The CFS-J is 12-item self-report questionnaire ("Appendix"). The original English-language version of the CFS was developed by Martin and Rubin [6] to assess three aspects of cognitive flexibility: (a) the awareness that options and alternatives are available in any given situation, (b) the willingness to be flexible and adapt to the situation, and (c) self-efficacy in being flexible. Responses are made on a 6-point Likert scale: 6 (strongly agree), 5 (agree), 4 (slightly agree), 3 (slightly disagree), 2 (disagree), and 1 (strongly disagree). Items 2, 3, 5, and 10 of the scale are reverse-coded. Total scores range from 12 to 72, with higher scores indicating better cognitive flexibility (see Table 2 for the content of the original questionnaire). The original authors [6] and successive study [16] have shown that it has good concurrent, construct, and criterion-related validity when used with college student samples. The factor structure of the original CFS was reported in neither the original study [6], nor successive study [16], but Dennis and Vander Wal [7] reported that the scale has a one-factor structure, although no evidence for this was provided. The CFS has also been translated into Turkish and confirmed to be valid and reliable [17].

#### Cognitive flexibility inventory-Japanese version (CFI-J)

The CFI [7] is a 20-item, two-subscale self-report questionnaire, to which responses are made on a 7-point Likert scale ranged from 1 = “strongly disagree” to 7 = “strongly agree”. The CFI is designed to assess cognitive flexibility in relation to thinking adaptively, rather than maladaptively, when encountering stressful life events. The “alternative” subscale (10 items; α = 0.91) reflects a person’s ability to generate multiple solutions to difficult situations and perceive multiple alternative explanations of events; the “control” subscale (six items; α = 0.84–0.86) reflects a person’s tendency to perceive difficult situations as being controllable. Higher scores on the CFI are indicative of higher cognitive flexibility. The reliability and validity of the CFI-J’s alternative (α = 0.88) and control (α = 0.77) subscales have been confirmed by Tokuyoshi and Iwasaki [18].

#### Cognitive control scale (CCS)

Cognitive control refers to processes that allow information processing and behavior to vary adaptively from moment to moment depending on current goals, rather than remaining rigid and inflexible [19]. The CCS [20] is an 11-item Japanese language self-report questionnaire designed to assess the ability to voluntarily use cognitive behavioral therapy-like skills in daily life, and is based on Freeman’s cognitive skills [21]. Responses to the CCS are scored on a 4-point Likert scale ranging from 1 = “never” to 4 = “most surely”. The CCS (α = 0.81) comprises two subscales: “logical analysis” (six items; α = 0.79), which measures active and objective problem-solving skills, and “refraining from catastrophic thinking” (five items; α = 0.72), which measures the ability to be detached from negative thinking to alleviate catastrophic cognitions.

#### Automatic thought questionnaire-revised-short version in Japanese (ATQ-R-JS)

The ATQ-R [22] is a 40-item self-report questionnaire comprising 10 positive and 30 negative items that appear in the original ATQ, which was developed by Hollon and Kendall [23] to assess the extent of an individual’s experience of negative and positive automatic thoughts. Responses are made on a 5-point Likert scale ranging from 1 = “not at all” to 5 = “all the time”. Because the 40-item ATQ-R is not considered an ideal instrument for use in clinical situations, Sakamoto et al. [24] developed a short version in Japanese (ATQ-R-JS), which comprises two subscales of negative (six items; α = 0.88) and positive (six items; α = 0.82) automatic thoughts.

### Statistical analyses

Cronbach’s alpha and McDonald’s omega coefficients were used to examine the internal consistency of the CFS-J, and Spearman’s correlation coefficient and intra-class correlation coefficients (ICC) were used to establish the test–retest reliability over an interval of 1–2 weeks. The scale’s concurrent validity was assessed through calculating correlations with related measures, comprising the CFI-J, CCS, and ATQ-R-JS. We performed an exploratory factor analysis (EFA) to examine the factor structure of the CFS, using the maximum likelihood method with promax rotation and Kaiser’s normalization. We used the following criteria to determine the optimal number of factors: Kaiser’s eigenvalues [25], parallel analysis [26], and minimum average partials (MAP) [27]. Factor loadings of 0.4 or greater were considered to be acceptable [28]. All data were analyzed using SPSS for Windows (version 15) and R (version 3.1.3).

## Results

### Respondents’ demographic characteristics and total score distributions

The demographic characteristics of the eligible respondents (n = 335) and their scale score means and standard deviations are listed in Table 1. About 72 % were female. We do not have the exact distribution of participants’ ages because we collected this information as stratified data; however, all were aged 18 years or older and the median age stratum was 20 s. The mean CFS-J total score was 46.3, with a standard deviation of 7.7, minimum of 24, maximum of 71, kurtosis of 0.26, and skewness of 0.27, and the hypothesis of normal distribution was not supported (*p* = 0.047) by the Shapiro–Wilk test of normality.

**Table 1:** Demographic variables and the scale means

Variables	Samples		CFS-J total Score	
	N	%	Mean	S.D.
Total	335	100.0	46.3	7.7
Sex				
Male	94	28.1	49.1	8.1
Female	240	71.6	45.3	7.2
Unknown	1	0.3		
Age				
10s	115	34.3	44.0	7.3
20s	122	36.4	46.4	7.5
30s	36	10.7	49.0	6.1
40s	37	11.0	48.7	7.5
50s	19	5.7	50.5	8.5
60s	3	0.9	50.7	6.0
Unknown	3	0.9		
Occupation				
Office worker	51	15.2	48.5	8.1
Public service	45	13.4	48.9	6.7
Temporary worker	15	4.5	44.5	6.0
Student	217	64.8	45.3	7.6
Unknown	8	2.4		
Education				
High school	105	31.3	44.0	7.5
College	47	14.0	45.3	7.9
University	180	53.7	48.0	7.2
Unknown	3	0.9		

### Internal consistency

Cronbach’s alpha coefficient for the CFS-J was 0.847 and McDonald’s omega coefficient was 0.871, indicating the acceptable range for internal consistency [29]. When the alpha coefficient for each of the 12 items was calculated by successively eliminating each item, results ranged from 0.819 to 0.852. Simultaneously eliminating items 2, 5, and 11 improved the internal consistency substantially to 0.863 (omega = 0.888), and the means (SD) of the nine-item CFS-J were 34.7 (6.4), 37.0 (6.6), and 33.9 (6.0) for total scores, men, and women, respectively. The correlation between scores on the 12- and nine-item versions was significantly high at 0.969.

### Test–retest reliability

The test–retest reliability assessment was conducted with 107 eligible responders (33 men and 74 women). Mean (SD) scores on the CFS-J were 47.1 (7.4) for the total sample, 48.9 (7.4) for men, and 46.3 (6.9) for women. The test–retest correlation had a moderate to strong range (Spearman’s = 0.687, ICC = 0.689). When items 2, 5, and 11 were removed, the correlation for the nine-item CFS-J substantially improved (Spearman’s = 0.692, ICC = 0.709).

### Factorial validity

The Kaiser–Meyer–Olkin (KMO) measure of sampling adequacy [30] for the 12 item scale was 0.880, indicating meritorious sampling adequacy [31], and Bartlett’s test of sphericity was significant (*p* < *0*.001). The KMO increased to 0.882 in the nine-item CFS-J after items 2, 5, and 11 were eliminated. The eigenvalues of the first five factors in the 12-item CFS-J were 4.83, 1.09, 0.99, 0.88, and 0.79, in descending order, accounting for 40.3, 9.1, 8.3, 7.4, and 6.6 % of the variance, respectively. Kaiser’s criterion with eigenvalues over 1.0 supported a two-factor solution. The MAP values for the first three principal components of the CFS-J item correlation matrix were 0.018, 0.029, and 0.044, respectively, thus indicating a one-factor solution. The first three eigenvalues of simulated components by the parallel analysis were 1.32, 1.24, and 1.17, thus indicating a one-factor solution. In the nine-item CFS-J, the eigenvalues of the first three factors were 4.41, 0.84, and 0.76, accounting for 49.0, 9.3, and 8.2 % of the variance, respectively, thus Kaiser’s criterion with eigenvalues over 1.0 supported a one-factor solution, and the MAP and the parallel analysis also supported a one-factor solution.

EFA was used to examine the factor structure because a two-factor solution was possible, however, exhaustive analyses, including popular oblique rotations other than the promax method, could not adequately resolve the two-factor model. Thus, the factor loadings for the one-factor model only are shown in Table 2. It can be seen from the table that items 2, 5, and 11 had relatively smaller loadings on the factor, whereas all others had an acceptable level of 0.4 or more. The results of the one-factor model for the nine-item version of the scale, after eliminating these three items, are also shown in Table 2. The factor loadings of all items were 0.4 or more. The two right-hand columns show the Pearson correlation coefficients between each item and the rest of the total CFS-J score. All items indicated significant results based on the null hypothesis test for correlation coefficients (*p* < 0.001).

**Table 2:** Exploratory factor analysis of the CFS-J

Item no.	Items marked (R) are reverse scored	Factor loading/communality				Correlation of item-rest scores	
		12-item		9-item		12-item	9-item
1	I can communicate an idea in many ways	*0.688*	0.474	*0.691*	0.478	*0.619*	*0.633*
2	I avoid new and unusual situations (R)	0.367	0.135	NA	NA	0.345	NA
3	I feel like I never get to make decisions. (R)	*0.598*	0.357	*0.589*	0.348	*0.559*	*0.545*
4	I can find workable solutions to seemingly unsolvable problems	*0.692*	0.479	*0.700*	0.491	*0.620*	*0.648*
5	I seldom have choices when deciding how to behave (R)	0.298	0.009	NA	NA	0.294	NA
6	I am willing to work at creative solutions to problems	*0.596*	0.355	*0.593*	0.352	*0.542*	*0.556*
7	In any given situation, I am able to act appropriately	*0.622*	0.387	*0.641*	0.410	*0.533*	*0.569*
8	My behavior is a result of conscious decisions that I make	*0.535*	0.286	*0.535*	0.286	*0.494*	*0.502*
9	I have many possible ways of behaving in any given situation	*0.768*	0.590	*0.783*	0.613	*0.683*	*0.714*
10	I have difficulty using my knowledge on a given topic in real life situations (R)	*0.540*	0.292	*0.529*	0.280	*0.538*	*0.518*
11	I am willing to listen and consider alternative for handling a problem	*0.416*	0.173	NA	NA	0.395	NA
12	I have the self-confident necessary to try different way of behaving	*0.799*	0.639	*0.780*	0.609	*0.744*	*0.717*
	% of variance	*35.5*		*42.9*			

Italics characters represent factor loadings >0.40, or correlation >0.4

### Concurrent validity

Table 3 shows the Pearson correlation coefficients between the CFS-J (12- and nine-item versions) and the CFI-J, CCS, and ATQ-R-JS scales, as well as the results of the null hypothesis test. The CFS-J had a significantly positive correlation with the CFI-J and its alternative and control subscales, with the CCS, and with the positive subscale of the ATQ-R-JS. Further, it had a significant negative correlation with the negative subscale of the ATQ-R-JS.

**Table 3:** Pearson’s correlation coefficient of CFS-J with other related scales and the null hypothesis tests

	CFI-J	CFI-J_A	CFI-J_C	CCS	ATQ-R-Pos	ATQ-R-Neg
CFS (12-item)	0.760***	0.539***	0.668****	0.650***	0.441***	−0.534***
CFS (9-item)	0.721***	0.484***	0.668***	0.645***	0.423***	−0.534***

*** p < 0.001 (two-tailed): null hypothesis test for coefficient of correlation

## Discussion

### Reliability and validity

Cronbach’s alpha and McDonald’s omega coefficients in the total sample of the 12- and nine-item versions of the CFS-J exceeded 0.8, which indicates that the scale has good internal consistency. The test–retest reliability, as measured by Spearman’s coefficient of correlation and ICC, which were moderate for the 12-item and good for the nine-item scale versions, was also satisfactory.

EFA provided evidence that the 12- and nine-item versions of the CFS-J have a one-factor structure, although Kaiser’s criterion with eigenvalues over 1.0 suggested the possibility of a two-factor solution. Even with a one-factor solution, the factor accounted for 35.5 % of the variance for the 12-item and 42.9 % for the nine-item versions of the scale, and all factor loadings were appropriate at 0.4 or more, except items 2 and 5 of the 12-item version. Therefore, a one-factor solution adequately explained the factor structure of the CFS-J.

The concurrent validity of the CFS-J was confirmed by correlations between scores on the CFS-J and those on other related measures. CFS-J scores were significantly and positively related with those on the CFI-J, indicating that a person with a higher CFS-J score is likely to also have a higher CFI-J score. Further, cognitive flexibility, as assessed by the CFS-J, was significantly and positively associated with cognitive control skills, as assessed by the CCS. Correlations with the positive and negative subscales of the ATQ-R-JS indicated that greater cognitive flexibility, as measured by the CFS-J, was significantly associated with increased positive and decreased negative automatic thoughts, respectively. These results are consistent with those of Johnco [1], who stated that “these processes seem important for the successful implementation of cognitive restructuring, where the individual is required to identify a negative automatic thought, generate evidence that contradicts that thought, and subsequently generate a more adaptive or helpful way of interpreting the situation.” In this way, we established the concurrent validity of the CFS-J.

### Appropriate version of the CFS-J for use in future studies

The internal consistency (Cronbach’s alpha and McDonald’s omega) results suggested that the nine-item version of the CFS-J was superior to the 12-item version; however, the internal consistency (alpha = 0.847, omega = 0.871) and test–retest reliability (0.687) of the 12-item version were also more than acceptable, which suggests that both versions are adequate for use in future psychological assessment. Most factor loadings were more than adequate at 0.4. The correlation coefficients between the 12-item version and the other assessed scales were generally higher than those for the nine-item version, and the correlation between scores on the 12- and nine-item versions was significantly high. Moreover, there is no substantial difference in burden of testing time between the two versions, and the result of 12-item version is internationally comparable with other language versions. On the basis of these considerations, we recommend using the 12-item version of the CFS-J for conducting research and assessments in future studies.

### Limitations and future research directions

The unique contributions of this paper are that we translated the CFS, which is in common use in the English language as a tool to assess cognitive flexibility, into Japanese and then confirmed its reliability and validity. However, the results of this study have some potential limitations. First, generalization of the results should be done with caution because there were more women than men participants, they were recruited from one specific area in Japan and aged 18 years or older, and most were aged in their 20 s. In future studies, it will be necessary to examine whether the developed measure is suitable for use with samples of different demographic types. Second, the participants comprised a nonclinical sample. Future studies should assess the reliability and validity of the CFS-J using clinical samples, including patients with depression, an anxiety disorder, posttraumatic stress disorder, or an eating disorder, given that the English version of CFS has been applied and demonstrated to have potential effectiveness in assessing the outcomes of treatment for this population.

## Conclusions

We developed the Japanese version of the CFS and confirmed its internal consistency, test–retest reliability, and concurrent validity using nonclinical samples. The translation processes followed the standard practice of translation and back-translation, including approval of correspondence of the translated items by the original scale’s author. Results indicated that the CFS-J has good internal consistency and acceptable test–retest reliability over the space of –2 weeks, and that it has a one-factor structure with adequate factor loadings. The CFS-J also showed significant correlations with other concurrent measures, including CFI-J, CSS, and ATQ-R-JS. Thus, the CFS-J has good reliability and validity as an instrument for assessing cognitive flexibility in nonclinical samples.

## Abbreviations

ATQ-R: automatic thought questionnaire-revised
ATQ-R-JS: automatic thought questionnaire-revised-short version in Japanese
CCS: cognitive control scale
CFI-J: cognitive flexibility inventory-Japanese
CFS: cognitive flexibility scale
CFS-J: cognitive flexibility scale-Japanese
EFA: exploratory factor analysis
ICC: intra-class correlation coefficients
KMO: Kaiser-Meyer-Olkin measure of sampling adequacy
MAP: minimum average partials
SD: standard deviation
